# Preoperative Adult-Type Diffuse Glioma Subtype Prediction with Dynamic Contrast-Enhanced MR Imaging and Diffusion Weighted Imaging in Tumor Cores and Peritumoral Tissue—A Standardized Multicenter Study

**DOI:** 10.3390/diagnostics15050532

**Published:** 2025-02-21

**Authors:** Leonie Zerweck, Uwe Klose, Urs Würtemberger, Vivien Richter, Thomas Nägele, Georg Gohla, Kathrin Grundmann-Hauser, Arne Estler, Christer Ruff, Gunter Erb, Ulrike Ernemann, Till-Karsten Hauser

**Affiliations:** 1 Department of Diagnostic and Interventional Neuroradiology, University Hospital Tuebingen, 72076 Tuebingen, Germanytill-karsten.hauser@med.uni-tuebingen.de (T.-K.H.); 2 Department of Neuroradiology, Medical Center, Faculty of Medicine, University of Freiburg, 79106 Freiburg im Breisgau, Germany; 3 Institute of Medical Genetics and Applied Genomics, University Hospital Tuebingen, 72076 Tuebingen, Germany; 4 Bracco Group, Medical and Regulatory Affairs, 78467 Konstanz, Germany

**Keywords:** glioma, glioblastoma, astrocytoma, oligodendroglioma, diffusion kurtosis imaging, dynamic contrast-enhanced MRI

## Abstract

**Background/Objectives**: The non-invasive identification of glioma subtypes is useful for initial diagnosis, treatment planning, and follow-up. The aim of this study was to evaluate the performance of diffusion kurtosis imaging (DKI) and dynamic contrast-enhanced (DCE)-MRI in differentiating subtypes of adult-type diffuse gliomas. **Methods**: In a prospective multicenter study, standardized MRI was analyzed in 59 patients with adult-type diffuse glioma. DKI and DCE-MRI parameter values were quantitatively evaluated in ROIs of contrast-enhancing/solid tumor and four concentric shells of peritumoral tissue. The parameter means of glioblastomas, IDH wildtype; astrocytomas, IDH mutant; and oligodendrogliomas, IDH mutant were compared. Binary logistic regression analyses were performed to differentiate between IDH mutant and IDH wildtype gliomas and between IDH mutant astrocytomas and oligodendrogliomas. ROC curves were analyzed for each parameter and for combined regression. **Results**: Significant differences between the three aforementioned subtypes were found for the DKI and DCE-MRI parameters, depending on the distance to the tumor core. A combination of the parameters’ apparent diffusion coefficient (ADC) and fractional volume of extravascular extracellular space (ve) revealed the best prediction of IDH mutant vs. wildtype gliomas (AUC = 0.976 (0.943–1.000)) and astrocytomas vs. oligodendrogliomas (AUC = 0.840 (0.645–1.000)) with the lowest Akaike information criterion. **Conclusions**: The combined evaluation of DKI and DCE-MRI at different distances to the contrast-enhancing/solid tumor seems to be helpful in predicting glioma subtypes according to the WHO 2021 classification.

## 1. Introduction

The classification of gliomas and the identification of their subtypes based on the World Health Organization (WHO) 2021 classification of central nervous system (CNS) tumors are imperative for predicting patients’ prognoses and determining the most suitable treatment strategies [1]. Accurate tumor subtyping relies on the histopathological and molecular features of tumor biopsies [2,3]. Nevertheless, a reliable non-invasive method for tumor evaluation is crucial during follow-up, particularly when patients are not eligible for surgery or when patients are monitored for potential tumor recurrence [4]. Magnetic resonance imaging (MRI) remains the preferred imaging technique for initial diagnosis, treatment planning, and follow-up examinations, giving information about the tumor’s location, extent, and interaction with the adjacent tissue [5,6,7].

In the MRI of gliomas, perfusion-weighted imaging (PWI) is commonly used [8,9,10,11]. Dynamic contrast-enhanced (DCE) MRI is a PWI technique that provides reliable quantification of the blood–brain barrier and the microvascular permeability with high spatial resolution [8,12].

Diffusion-weighted imaging (DWI) is also a common component of the neurooncological MRI protocol [10,11,13,14]. Conventional diffusion-weighted imaging (DWI) operates under the premise that water molecule diffusion follows a Gaussian distribution [3,14,15]. However, in biological tissues, cell membranes and compartments lead to non-Gaussian diffusion patterns [3,14,15]. Diffusion kurtosis imaging (DKI) is a complementary diffusion tensor imaging (DTI) technique that moves beyond the Gaussian assumption and quantifies the deviation from Gaussian behavior [16]. By providing deeper microstructural information, DKI has shown potential for improved glioma grading compared to standard diffusion metrics and conventional morphological imaging [17,18,19].

Recently, the combined evaluation of DCE-MRI and DKI was proposed to facilitate the prediction of high-grade (HGG) vs. low-grade gliomas (LGG), IDH1/2 wildtype vs. IDH1/2 mutant gliomas, and high-grade oligodendroglial vs. high-grade astrocytic gliomas [20].

The objective of this study was to quantitatively compare the diagnostic performance of DCE-MRI and DKI in molecular subtype identification according to the WHO 2021 classification of CNS tumors, which divides adult-type diffuse gliomas into glioblastomas, IDH wildtype; astrocytomas, IDH mutant; and oligodendrogliomas, IDH mutant and 1p/19q-codeleted [2,21,22]. We aimed to not only compare the parameters in the tumor center but also in the peritumoral tissue.

## 2. Materials and Methods

### 2.1. Study Design

A prospective multicenter study was conducted at six neurosurgical centers in China. The study adhered to the principles of the Declaration of Helsinki and was approved by the local ethics committees at each center (ethical codes: YW2016-003-02, YW2016-003-02, Y55-2, 20160406-4, B2017-049-01, 2016-Y-004-02; see also: Institutional Review Board Statement). All participants gave written informed consent.

### 2.2. Patients

A total of 108 patients were enrolled and underwent standardized MR imaging. Inclusion criteria were the presence of a suspected supratentorial adult-type diffuse glioma and a planned tumor biopsy/surgery with histopathologic analysis performed within four weeks after the study MRI. Patients were excluded if they had contraindications to MRI (e.g., pacemakers, metal implants, severe claustrophobia), allergy to contrast agents, severe renal impairment with GFR/eGFR < 30 mL/min, or if they had received radiotherapy or chemotherapy prior to the biopsy or surgery. Further exclusion criterion was insufficient image quality due to poor patient compliance during MRI and resulting movement artifacts.

### 2.3. MR Imaging

All MRI examinations were performed using a Siemens-manufactured 3T MRI scanner to ensure comparability across all sites. The head coils employed were those used in clinical practice (usually 20-channel head coils).

The MR imaging protocol included conventional MRI sequences (axial T1 spin echo (SE)/fast spin echo (FSE) sequences before and after contrast administration, axial T2 FLAIR, and post-contrast 3D-T1 gradient echo (GRE) imaging).

DCE-MRI was acquired with a dynamic 3D-T1 volumetric interpolated breath-hold examination (VIBE) sequence (TR 4 ms, TE 1.8 ms, voxel size 1.5 × 1.1 × 4.0 mm, number of excitations (NEX) 1, and parallel acquisition technique (PAT) 2). Three flip angles (FA) (6°, 9°, 15°) were applied for the T1 quantification. Subsequent to the administration of the contrast agent (Gadobenate dimeglumine (MultiHance; Bracco)) at a single dose of 0.1 mmol/kg and an injection rate of 4 mL/s, dynamic measurements were conducted.

DWI was performed using a 2D echo-planar imaging (EPI) sequence with multiple b-values (0, 500, 1000, 1500, 2000, 2500 s/mm^2^) for the DKI analysis (TR 5900 ms, TE 95 ms, voxel size 2.0 × 2.0 × 5.0 mm, number of diffusion encoding directions 6/b value, EPI factor 128).

### 2.4. Image Analysis

The post-processing and analysis of MRI data were carried out off-site at the imaging core lab.

The following DCE-MRI parameters were based on the extended Tofts model: Volume transfer constant (Ktrans), reflux exchange rate (Kep), fractional volume of plasma (vp), fractional volume of extravascular extracellular space (ve), cerebral blood volume (CBV), time to peak (TTP), peak, area under the curve (AUC), wash in, and wash out.

The DWI parameter values’ apparent diffusion coefficient (ADC) and mean kurtosis (MK) were calculated using scripts written in MATLAB (R2018b (The MathWorks, Inc., Natick, MA, USA)). We focused on the DKI parameter MK because a recent evaluation showed promising results in the diagnostics of adult-type diffuse gliomas [20].

**Figure 1:**
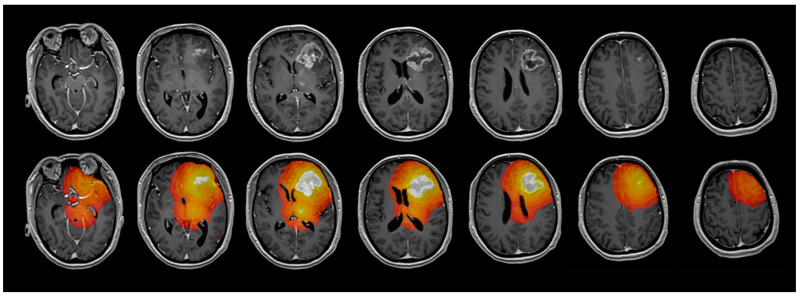
Exemplary maps of one patient with glioblastoma in the left frontal lobe. The contrast-enhancing tumor (ROI_1_) and 4 concentric ROIs (ROI_2–5_) with a distance of 5 mm between each ROI were evaluated.

**Figure 2:**
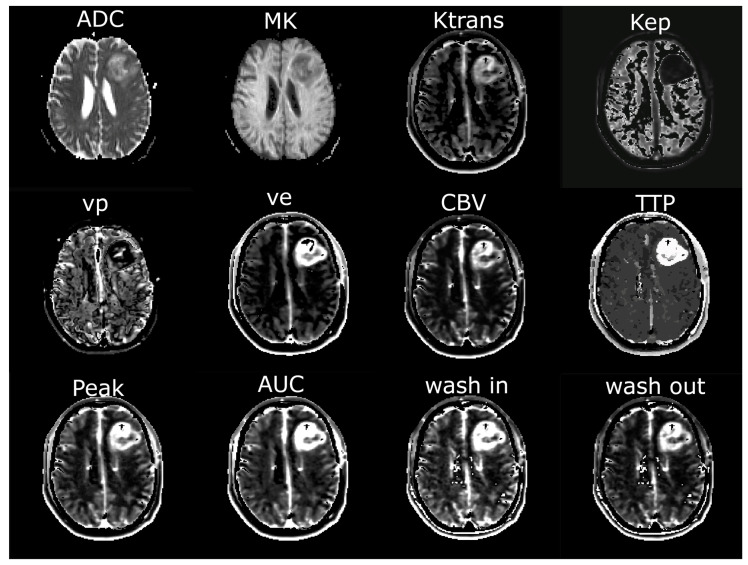
Exemplary maps of each image parameter of one patient with glioblastoma in the left frontal lobe. ADC = apparent diffusion coefficient; MK = mean kurtosis; Ktrans = volume transfer constant; Kep = reflux exchange rate; vp = fractional volume of plasma; ve = fractional volume of extravascular extracellular space; CBV = cerebral blood volume; TTP = time to peak; AUC = area under the curve.

For further analysis, regions of interest (ROI) were defined using the anatomical pre- and post-contrast T1, FLAIR, and T2 images. Both tumors with contrast-enhancing lesions and non-enhancing tumors were evaluated. Contrast-enhancing tumors were segmented into contrast-enhancing tumor tissue (ROI_1_), necrosis, and non-enhancing tumor tissue/perifocal edema using the MONAI framework [23] with the Multimodal Brain Tumor Segmentation Challenge (BraTS) Glioma model. Non-enhancing tumors were segmented with the BraTS PED model. The MONAI slicer plugin was implemented in Tensorflow using the BraTS 2018 training data set, which consists of 285 hand-labeled cases. The model performed best in the 2018 BraTS challenge, reaching the following dice scores: contrast-enhancing tumor: 0.8145; whole tumor: 0.9068; and necrotic tumor core: 0.8596 [24]. The results of the segmentation were verified through visual inspection. Five ROIs (ROI_2_–ROI_6_) were defined in shells around the central ROI_1_, with a distance of 5 mm between each of the shells. Necrotic tissue and cystic cavities were excluded from the analysis, so all ROIs exclusively contained brain tissue. CSF spaces and bones were systematically excluded from the analysis. Exemplary maps of the ROI selection are shown in Figure 1. ROI_6_, where nearly physiological tissue was expected, was not evaluated in the further analysis, but served to normalize the DCE-MRI parameter values of ROI_1_–ROI_5_ by calculating the ratio of the parameter values in ROI_1–5_/ROI_6_. Figure 2 illustrates the exemplary maps of each evaluated parameter.

### 2.5. Postoperative Tumor Subtyping

A review of the postoperative pathology reports was conducted to determine the histopathologic diagnosis according to the 2021 WHO classification of CNS tumors [2]. We distinguished between the following [2,21]:

(i). Glioblastomas, IDH wildtype;
(ii). Astrocytomas, IDH mutant;
(iii). Oligodendrogliomas, IDH mutant and 1p/19q-codeleted.

Gliomas, IDH wildtype, not elsewhere classified (NEC) were excluded from further analysis due to the expected small sample size.

Subgroups (ii) and (iii) included both LGG and HGG. The objective of the study was not to differentiate between LGG and HGG or to perform tumor grading but rather to distinguish between the subgroups (i)–(iii).

### 2.6. Statistical Analysis

Statistical analyses were performed using SPSS Statistics (IBM Corp. Released 2021. IBM SPSS1 Statistics for Windows, version 28.0. Armonk, NY, USA: IBM Corp). Figures with statistical content were generated with SPSS Statistics and MATLAB.

Because homogeneity of variance was not present in all cases, Welch analysis of variance (ANOVA) was conducted to test for differences between (i) glioblastomas, IDH wildtype; (ii) astrocytomas, IDH mutant; and (iii) oligodendrogliomas, IDH mutant in each ROI_1-5_. Games–Howell post hoc tests were performed.

The performance of all DCE-MRI and DKI parameters to discriminate between the aforementioned groups (i)–(iii) was analyzed. For this purpose, univariate binary logistic regression analyses were performed to assess the diagnostic value of all parameters in each ROI to differentiate between (i) IDH wildtype vs. IDH mutant gliomas and (ii) astrocytomas vs. oligodendrogliomas. Receiver operating characteristic (ROC) curves were generated for each parameter to determine the area under the curve (AUC), sensitivity, and specificity. The results with the highest Youden index were defined as the optimal cutoff value.

Multivariant binary logistic regression analyses were then performed with the distinction between (i) IDH wildtype vs. IDH mutant gliomas and (ii) astrocytomas vs. oligodendrogliomas as dependent variables and the DCE-MRI parameter and the DKI parameter with the highest AUC of all ROIs as the independent variables. The Akaike information criterion (AIC) was used to account for the number of predictors.

The performance of the models with combined DCE-MRI and DKI parameters were compared to the individual parameters using DeLong tests.

The alpha level of each test was *p* < 0.05, with false discovery rate (FDR) used to correct for multiple comparisons.

## 3. Results

### 3.1. Patients

One hundred and eight patients with suspected cerebral glioma were enrolled. After histopathologic and molecular review, 84 patients with adult-type diffuse supratentorial gliomas WHO grade 2–4 were included in the further analysis. Data sets of 25 patients were excluded due to poor patient compliance, resulting in movement artifacts and insufficient image quality, or due to erroneous MRI data acquisition with false MRI parameter settings (14 glioblastomas, IDH wildtype (WHO grade 4); 4 astrocytoma, IDH mutant (WHO grade 3); 2 astrocytoma, IDH mutant (WHO grade 2); 5 oligodendrogliomas, IDH mutant (WHO grade 3)). A total of 59 data sets were included in the final analysis. General patient characteristics are shown in Table 1.

### 3.2. Comparison of DKI and DCE-MRI Parameters in Each ROI

**Figure 3:**
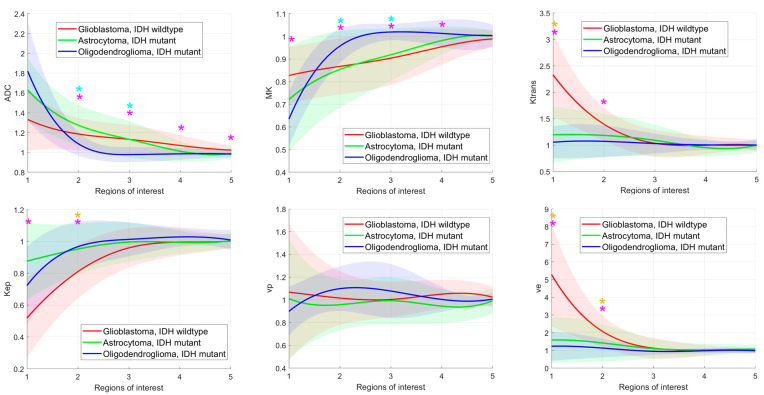

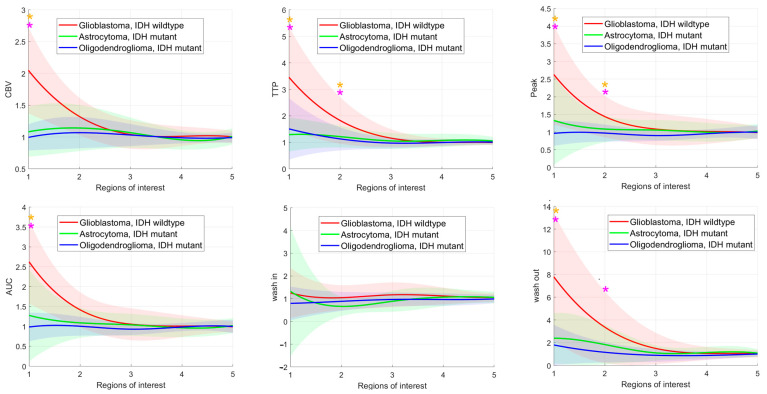
Glioma subgroup comparisons of the apparent diffusion coefficient (ADC), mean kurtosis (MK), and the dynamic contrast-enhanced (DCE) MRI parameter volume transfer constant (Ktrans), reflux exchange rate (Kep), fractional volume of plasma (vp), fractional volume of extravascular extracellular space (ve), cerebral blood volume (CBV), time to peak (TTP), peak, area under the curve (AUC), wash in, and wash out. Values between regions of interest are interpolated. Light bars represent the mean values ± the standard deviation of each parameter. Significant differences are marked with an asterisk (*) above the graphs (yellow *: differences between glioblastomas, IDH mutant and astrocytomas, IDH mutant; turquoise *: differences between astrocytomas, IDH mutant and oligodendrogliomas, IDH mutant; pink *: differences between glioblastomas, IDH wildtype and astrocytomas, IDH mutant).

The means and standard deviations of the DKI and DCE-MRI parameters of all glioma subgroups in each ROI are shown in Figure 3. ADC was significantly different between glioblastomas, IDH wildtype and oligodendrogliomas, IDH mutant in ROI_2_–ROI_5_ and significantly different between astrocytomas, IDH mutant and oligodendrogliomas, IDH mutant in ROI_2_ and ROI_3._ MK differed significantly between glioblastomas, IDH wildtype and oligodendrogliomas, IDH mutant in ROI_1_–ROI_4_ and between astrocytomas, IDH mutant and oligodendrogliomas, IDH mutant in ROI_2_ and ROI_3._ Almost all DCE-MRI parameters (Ktrans, Kep, ve, CBV, TTP, peak, AUC, wash out) revealed group differences with significant post hoc test results between glioblastomas, IDH wildtype and astrocytomas, IDH mutant and between glioblastomas, IDH wildtype and oligodendrogliomas, IDH mutant in ROI_1_ and partially in ROI_2._

### 3.3. Diagnostic Performance in Differentiating IDH Wildttype from IDH Mutant Gliomas

**Figure 4:**
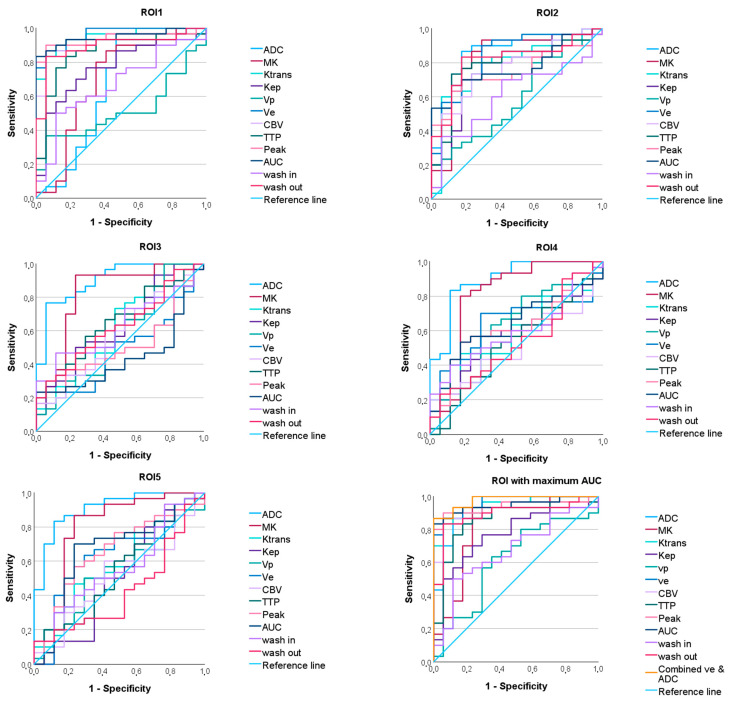
ROC curves predicting the IDH mutation status of the apparent diffusion coefficient (ADC), mean kurtosis (MK), and the dynamic contrast-enhanced (DCE) MRI parameter volume transfer constant (Ktrans), reflux exchange rate (Kep), fractional volume of plasma (vp), fractional volume of extravascular extracellular space (ve), cerebral blood volume (CBV), time to peak (TTP), peak, area under the curve (AUC), wash in, and wash out in all regions of interest (ROI). On the bottom right, the maximum ROC of each parameter and the ROC of the combined parameters ve and ADC are shown.

**Figure 5:**
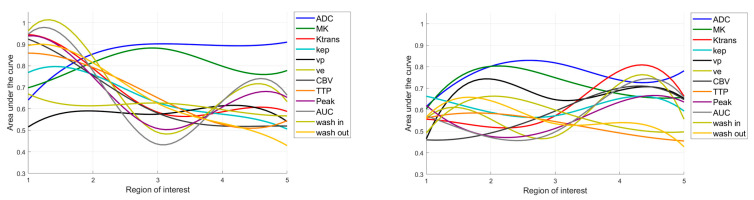
Area under the curve values predicting the IDH status (**left**) and differentiating astrocytomas vs. oligodendrogliomas (**right**), depending on the region of interest. ADC = apparent diffusion coefficient; MK = mean kurtosis; Ktrans = volume transfer constant; Kep = reflux exchange rate; vp = fractional volume of plasma; ve = fractional volume of extravascular extracellular space; CBV = cerebral blood volume; TTP = time to peak; AUC = area under the curve. When predicting the IDH status, the ROIs close to the core appear to be beneficial, while when differentiating between astrocytomas vs. oligodendrogliomas, the evaluation of the more distant ROIs becomes more important.

The ROC curve analysis revealed significant AUC values for the parameters ADC and MK and the DCE-MRI parameters Ktrans, Kep, ve, CBV, TTP, Peak, AUC, wash in, and wash out, with sensitivity, specificity, and AUC varying between the ROIs (see Figure 4, Table 2). While the DCE-MRI parameters revealed the highest AUC in ROI_1_, ADC and MK reached their maximum AUC in ROI_5_ and ROI_3_ (see Figure 5, Table 2).

Binary logistic regression with the DWI parameter with the maximum AUC (ADC in ROI_5_) and the DCE-MRI parameter with the maximum AUC (ve in ROI_1_) yielded a model with a lower AIC (18.527) than the individual parameters (ve: 26.297; ADC: 35.416). However, the AUC of the combined model was not significantly different from the AUC of the individual parameters (DeLong *p* = 0.444 vs. ve in ROI_1_, *p* = 0.077 vs. ADC in ROI_5_).

### 3.4. Diagnostic Performance in Differentiating Astocytomas, IDH Mutant from Oligodendrogliomas, IDH Mutant

**Figure 6:**
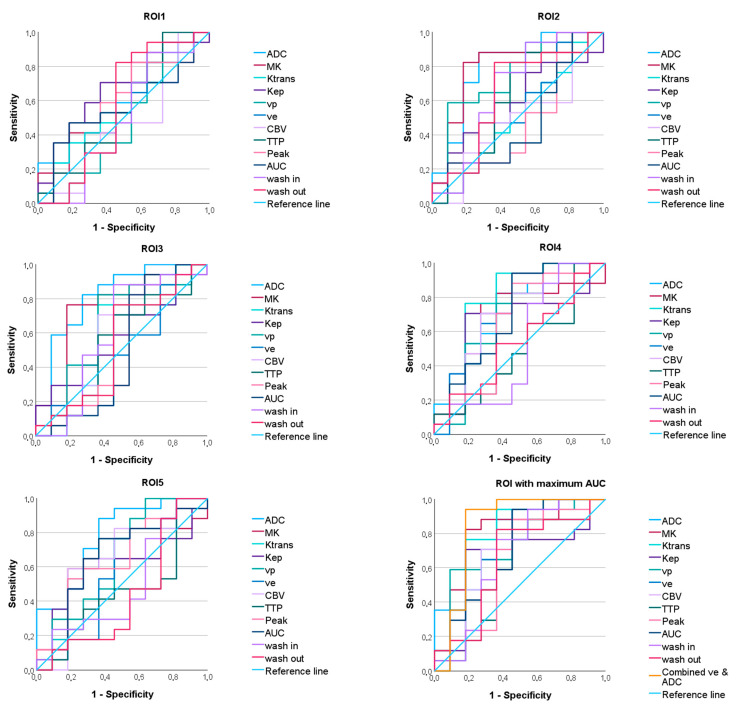
ROC curves for discriminating between astrocytomas, IDH mutant and oligodendrogliomas, IDH mutant of the apparent diffusion coefficient (ADC), mean kurtosis (MK), and the dynamic contrast-enhanced (DCE) MRI parameter volume transfer constant (Ktrans), reflux exchange rate (Kep), fractional volume of plasma (vp), fractional volume of extravascular extracellular space (ve), cerebral blood volume (CBV), time to peak (TTP), peak, area under the curve (AUC), wash in, and wash out in all regions of interest (ROI). On the bottom right, the maximum ROC of each parameter and the ROC of the combined parameters ve and ADC are given.

Significant AUC values were found for the parameters ADC, MK, Ktrans, cp, and ve (see Figure 6, Table 3). The AUC values differed depending on the ROI (see Figure 5).

The model obtained from the binary logistic regression analysis with the DWI parameter ADC (in ROI_3_) and the DCE-MRI parameter ve (in ROI_4_) showed a lower AIC (29.123) than the individual parameters (ve: 38.804; ADC: 29.474). However, the AUC of the combined model did not differ significantly from the AUC of the individual parameters (DeLong *p* = 0.139 vs. ve in ROI_4_, *p* = 0.836 vs. ADC in ROI_3_).

## 4. Discussion

In this study, we explored the performance of the DWI and DCE-MRI parameters and combined approaches in glioma subtype identification according to the WHO 2021 classification of CNS tumors. We not only evaluated contrast-enhancing and non-contrast-enhancing solid tumor regions but also perilesional tissue at different distances from the tumor core.

First, we investigated whether DKI and DCE-MRI parameters differ between glioblastomas, IDH wildtype; astrocytomas, IDH mutant; and oligodendrogliomas, IDH mutant.

Glioblastomas showed significantly higher DCE-MRI permeability and perfusion parameters than astrocytomas and oligodendrogliomas. This was particularly evident in the solid and contrast-enhancing tumor core and, to a lesser extent, in the immediately adjacent tissue. Our findings are in accordance with previous studies, reporting higher permeability measured by DCE-MRI in glioblastomas, IDH mutant [25] or in HGG, where increased angiogenesis with atypical vascular anatomy, irregular blood flow, and higher vessel permeability are expected [6]. Significant differences between glioblastoma, IDH wildtype and other gliomas not only in the contrast-enhancing tumor but also in the infiltration zones have been described previously [26], in agreement with our results. We did not find any significant differences in DCE-MRI parameters between astrocytomas and oligodendrogliomas, although the ability of DCE-MRI to predict the 1p/19q codeletion is controversial in the literature [25,27].

In our study, in contrast to the DCE-MRI parameters, the DWI parameters ADC and MK showed significant differences between oligodendrogliomas and glioblastomas and between oligodendrogliomas and astrocytomas. The differences were significant even at greater distances from the tumor core. Consistent with previous studies [13,21], we detected lower ADC values in IDH wildtype gliomas than in IDH mutant gliomas, as a surrogate for higher tumor cellularity, although this difference was not significant in the tumor core. We measured non-significantly lower ADC values in oligodendrogliomas than in astrocytomas in the tumor core, in agreement with previous findings [13,28]. Interestingly, in our study, this ratio changed significantly in the peritumoral tissue in the periphery.

In the second part of our study, we compared the diagnostic accuracy of all individual DKI and DCE-MRI parameters to differentiate between IDH wildtype and IDH mutant gliomas and between astrocytomas and oligodendrogliomas, as these comparisons allow the classification of gliomas into the three subgroups. Both, in the discrimination of the IDH mutation status and in the discrimination between astrocytomas and oligodendrogliomas, the ADC revealed the highest AUC of the DWI parameters and ve showed the highest AUC of all DCE-MRI parameters, when the parameters were analyzed at different distances to the tumor core. Therefore, we performed binary logistic regression analyses with ve and ADC as independent variables. In both comparisons, we obtained models with a lower AIC than that of the individual parameters, which were thus better predictors of the IDH status and the discrimination between astrocytomas and oligodendrogliomas, even when considering the larger number of variables. The AUC of the models was greater than the AUC of the individual parameters, although the differences were not significant.

It should be noted that due to the limited sample size in our study, the combined model was not validated in an independent control group. Therefore, it was not possible to quantify the extent to which the model was overfitted to our data. The generalizability of the results may also be limited because gliomas, IDH wild-type, NEC had to be excluded from further analysis due to low prevalence, making statistical analysis impossible. Nevertheless, we consider it very promising that the combined evaluation of the DKI and DCE-MRI parameters leads to better subgroup identification and that it is advantageous to analyze the parameters not only in the tumor core but also in the peritumoral tissue. Previous studies have already shown that combined approaches of DKI and DCE-MRI lead to a better differentiation between HGG and LGG [20,29,30], IDH mutant and wildtype gliomas [20,30,31], and 1p/19q codeleted and 1p/19q non-codeleted LGG [32], but these studies focused on a central tumor volume. Wang et al. recently analyzed DCE-MRI and DWI in three subregions of adult-type diffuse gliomas and found that combined approaches might facilitate the prediction of IDH genotype and prognosis [33], in line with our study, but they did not aim to differentiate between astrocytomas, IDH mutant and oligodendrogliomas, IDH mutant.

A novel finding of this study is that considering the tumor periphery leads to a significantly better prediction of the three glioma subtypes according to the WHO 2021 classification of CNS tumors.

Another limitation to our study was that secondary brain tumors were not evaluated. The study focused on glioma subtyping according to the IDH genotype and the 1p/19q codeletion status, but it did not consider other molecular biomarkers, such as o6-methylguanine DNA methyltransferase (MGMT).

In this study, the MONAI framework with the BraTS model was employed to segment the tumors and exclude necrotic tissue and cystic cavities. The results were then verified through visual inspection. However, it is plausible that certain components of the tissue were not completely excluded, potentially leading to the residual contamination of the results.

However, to the best of our knowledge, to date, no other prospective multicenter study has quantitatively compared the diagnostic performance of DKI and DCE-MRI in glioma subtyping according to the WHO 2021 classification in tumor cores and concentric shells of peritumoral tissue. Further studies with larger sample sizes and including gliomas, IDH wildtype, NEC, are recommended to validate our finding that suggests the combined evaluation of DWI and DCE-MRI in regions with different distances from to the tumor core leads to higher diagnostic accuracy.

## 5. Conclusions

This multicenter study revealed that the combined evaluation of the DWI parameter ADC and the DCE-MRI parameter ve show promise in predicting adult-type diffuse glioma subtypes. A further finding was that the combined evaluation of DWI and DCE-MRI parameters may be useful for the noninvasive identification of adult-type diffuse glioma subtypes, not only in tumor cores but also in peritumoral tissue, according to the WHO 2021 classification of CNS tumors.

## Figures and Tables

**Table 1:** General patient data.

Patients enrolled in the study	108
Patients excluded due to histopathological/molecular results	24
Patients excluded due to insufficient MRI quality	25
Patients included in the analysis	59
Mean age of the included patients ± standard deviation	45.3 ± 15.7
Female/Male ratio	1:1.6
Glioblastoma, IDH wildtype (WHO grade 4)	31 (47.4%)
Astrocytoma, IDH mutant (WHO grade 2)	12 (18.5%)
Astrocytoma, IDH mutant (WHO grade 3)	1 (1.5%)
Astrocytoma, IDH mutant (WHO grade 4)	4 (6.2%)
Oligodendroglioma, IDH mutant (WHO grade 2)	3 (4.6%)
Oligodendroglioma, IDH mutant (WHO grade 3)	8 (12.3%)

**Table 2:** Diagnostic performance of all diffusion kurtosis imaging and dynamic contrast-enhanced MRI parameters in differentiating between IDH mutant and IDH wildtype gliomas across all regions of interest (ROI).

		ROI_1_	ROI_2_	ROI_3_	ROI_4_	ROI_5_	ROI with Maximum AUC
**ADC**	**AUC** **(95% CI)**	0.641 (0.452–0.830)	0.855 * **(0.738–0.972)**	0.902 * **(0.812–0.992)**	0.894 * **(0.797–0.991)**	0.910 * **(0.824–0.995)**	0.910 * **(0.824–0.995)**
	**Sensitivity**	0.933	**0.867**	**0.767**	**0.833**	**0.833**	**0.833**
	**Specificity**	0.529	**0.824**	**0.941**	**0.882**	**0.882**	**0.882**
**MK**	**AUC** **(95% CI)**	0.698 * **(0.522–0.874)**	0.816 * **(0.672–0.959)**	0.882 * **(0.680–0.963)**	0.798 * **(0.638–0.958)**	0.778 * **(0.609–0.948)**	0.882 * **(0.680–0.963)**
	**Sensitivity**	**0.867**	**0.933**	**0.993**	**0.800**	**0.867**	**0.993**
	**Specificity**	**0.588**	**0.706**	**0.665**	**0.882**	**0.665**	**0.665**
**Ktrans**	**AUC** **(95% CI)**	0.945 * **(0.885–1.000)**	0.788 * **(0.648–0.928)**	0.584 (0.414–0.754)	0.586 (0.423–0.750)	0.588 (0.417–0.760)	0.945 * **(0.885–1.000)**
	**Sensitivity**	**0.833**	**0.600**	0.733	0.467	0.467	**0.833**
	**Specificity**	**0.941**	**0.941**	0.529	0.824	0.765	**0.941**
**Kep**	**AUC** **(95% CI)**	0.769 * **(0.628–0.909)**	0.759 * **(0.612–0.905)**	0.629 (0.463–0.796)	0.575 (0.409–0.740)	0.506 (0.321–0.690)	0.769 * **(0.628–0.909)**
	**Sensitivity**	**0.700**	**0.700**	0.500	0.567	0.800	**0.700**
	**Specificity**	**0.765**	**0.824**	0.765	0.706	0.353	**0.765**
**vp**	**AUC** **(95% CI)**	0.516 (0.350–0.681)	0.590 (0.422–0.758)	0.575 (0.398–0.751)	0.614 (0.443–0.784)	0.541 (0.371–0.711)	0.614 (0.443–0.784)
	**Sensitivity**	0.367	0.267	1.000	0.800	0.500	0.800
	**Specificity**	0.941	0.882	0.235	0.471	0.806	0.471
**ve**	**AUC** **(95% CI)**	0.963 * **(0.917–1.000)**	0.841 * **(0.721–0.961)**	0.490 (0.321–0.659)	0.637 (0.476–0.798)	0.633 (0.463–0.804)	0.963 * **(0.917–1.000)**
	**Sensitivity**	**0.867**	**0.900**	0.667	0.700	0.533	**0.867**
	**Specificity**	**0.941**	**0.706**	0.176	0.706	0.765	**0.941**
**CBV**	**AUC** **(95% CI)**	0.924 * **(0.848–1.000)**	0.755 * **(0.610–0.900)**	0.580 (0.411–0.750)	0.520 (0.353–0.686)	0.520 (0.347–0.692)	0.924 * **(0.848–1.000)**
	**Sensitivity**	**0.867**	**0.733**	0.600	0.200	0.600	**0.867**
	**Specificity**	**0.941**	**0.765**	0.529	1.000	0.588	**0.941**
**TTP**	**AUC** **(95% CI)**	0.859 * **(0.742–0.976)**	0.792 * **(0.654–0.930)**	0.651 (0.492–0.810)	0.527 (0.353–0.701)	0.545 (0.371–0.719)	0.859 * **(0.742–0.976)**
	**Sensitivity**	**0.833**	**0.733**	0.567	0.433	0.600	**0.833**
	**Specificity**	**0.824**	**0.882**	0.706	0.647	0.471	**0.824**
**Peak**	**AUC** **(95% CI)**	0.939 * **(0.868–1.000)**	0.749 * **(0.611–0.887)**	0.508 (0.340–0.676)	0.608 (0.444–0.772)	0.653 (0.487–0.819)	0.939 * **(0.868–1.000)**
	**Sensitivity**	**0.900**	**0.700**	0.500	0.533	0.567	**0.900**
	**Specificity**	**0.941**	**0.824**	0.294	0.824	0.765	**0.941**
**AUC**	**AUC** **(95% CI)**	0.947 * **(0.884–1.000)**	0.776 * **(0.645–0.908)**	0.435 (0.268–0.603)	0.635 (0.476–0.795)	0.663 (0.484–0.832)	0.947 * **(0.884–1.000)**
	**Sensitivity**	**0.833**	**0.633**	0.467	0.433	0.700	**0.833**
	**Specificity**	**1.000**	**0.882**	0.235	0.882	0.665	**1.000**
**Wash in**	**AUC** **(95% CI)**	0.669 * **(0.510–0.828)**	**0.614** **(0.451–0.777)**	0.627 (0.469–0.786)	0.596 (0.434–0.758)	0.567 (0.396–0.737)	0.669 * **(0.510–0.828)**
	**Sensitivity**	**0.500**	**0.700**	0.467	0.400	0.300	**0.500**
	**Specificity**	**0.824**	**0.588**	0.882	0.882	0.882	**0. 824**
**Wash out**	**AUC** **(95% CI)**	0.894 * **(0.798–0.991)**	0.814 * **(0.687–0.941)**	0.625 (0.464–0.787)	0.533 (0.362–0.705)	0.429 (0.258–0.6011)	0.894 * **(0.798–0.991)**
	**Sensitivity**	**0.833**	**0.833**	0.467	0.233	0.333	**0.833**
	**Specificity**	**0.941**	**0.824**	0.765	0.941	0.471	**0. 941**
**Combined ve and ADC**	**AUC** **(95% CI)**						0.976 * **(0.943–1.000)**
	**Sensitivity**						**0.933**
	**Specificity**						**0.882**

CI = confidence interval; ADC = apparent diffusion coefficient; MK = mean kurtosis; Ktrans = volume transfer constant; Kep = reflux exchange rate; vp = fractional volume of plasma; ve = fractional volume of extravascular extracellular space; CBV = cerebral blood volume; TTP = time to peak; AUC = area under the curve. ROI_1_ represents the tumor core and ROI_2-5_ represent concentric shells around ROI_1_, with increasing distance to ROI_1._ Significant AUC values are bold and marked with an asterisk (*).

**Table 3:** Diagnostic performance in differentiate between astrocytomas, IDH mutant and oligodendrogliomas, IDH mutant of all diffusion kurtosis imaging, and dynamic contrast-enhanced MRI parameters in all regions of interest (ROI).

		ROI_1_	ROI_2_	ROI_3_	ROI_4_	ROI_5_	ROI with Maximum AUC
**ADC**	**AUC** **(95% CI)**	0.615 (0.398–0.832)	0.802 * **(0.621–0.984)**	0.818 * **(0.649–0.988)**	0.738 * **(0.544–0.932)**	0.781 * **(0.600–0.961)**	0.818 * **(0.649–0.988)**
	**Sensitivity**	0.824	**0.882**	**0.824**	**0.824**	**0.882**	**0.824**
	**Specificity**	0.455	**0.455**	**0.727**	**0.636**	**0.636**	**0.727**
**MK**	**AUC** **(95% CI)**	0.604 (0.381–0.827)	0.802 * **(0.619–0.986)**	0.749 * **(0.547–0.950)**	**0.674** **(0.454–0.893)**	**0.658** **(0.442–0.874)**	0.802 * **(0.619–0.986)**
	**Sensitivity**	0.824	0.824	0.765	0.765	0.588	**0.824**
	**Specificity**	0.455	0.818	0.636	0.627	0.818	**0.818**
**Ktrans**	**AUC** **(95% CI)**	0.556 (0.334–0.778)	0.519 (0.290–0.747)	0.572 (0.330–0.814)	0.781 * **(0.576–0.968)**	0.663 (0.444–0.883)	0.781 * **(0.576–0.968)**
	**Sensitivity**	0.353	0.588	0.765	**0.941**	0.765	**0.941**
	**Specificity**	0.818	0.545	0.634	**0.636**	0.636	**0.636**
**Kep**	**AUC** **(95% CI)**	0.663 (0.456–0.871)	0.588 (0.373–0.804)	0.572 (0.356–0.788)	0.652 (0.429–0.876)	0.594 (0.377–0.810)	0.652 (0.429–0.876)
	**Sensitivity**	0.706	0.412	0.706	0.706	0.588	0.706
	**Specificity**	0.636	0.727	0.455	0.818	0.636	0.818
**vp**	**AUC** **(95% CI)**	0.465 (0.224–0.706)	0.743 * **(0.551–0.935)**	0.647 (0.418–0.867)	0.690 (0.467–0.912)	0.652 (0.428–0.877)	0.743 * **(0.551–0.935)**
	**Sensitivity**	0.941	**0.588**	0.824	0.765	1.000	**0.588**
	**Specificity**	0.273	**0.909**	0.636	0.636	0.364	**0.909**
**ve**	**AUC** **(95% CI)**	0.599 (0.380–0.818)	0.556 (0.327–0.785)	0.476 (0.238–0.713)	0.722 * **(0.510–0.933)**	0.556 (0.315–0.797)	0.722 * **(0.510–0.933)**
	**Sensitivity**	0.471	0.941	0.176	**0.941**	0.765	**0.941**
	**Specificity**	0.818	0.273	0.545	**0.545**	0.545	**0.545**
**CBV**	**AUC** **(95% CI)**	0.460 (0.228–0.692)	0.492 (0.261–0.723)	0.594 (0.354–0.833)	0.701 (0.487–0.914)	0.647 (0.417–0.877)	0.701 (0.487–0.914)
	**Sensitivity**	0.471	0.588	0.706	0.706	0.588	0.706
	**Specificity**	0.273	0.182	0.636	0.727	0.818	0.727
**TTP**	**AUC** **(95% CI)**	0.561 (0.328–0.795)	0.583 (0.344–0.822)	0.545 (0.312–0.779)	0.492 (0.263–0.721)	0.455 (0.225–0.685)	0.583 (0.344–0.822)
	**Sensitivity**	1.000	0.882	0.706	1.000	0.529	0.882
	**Specificity**	0.273	0.455	0.545	0.182	0.182	0.455
**Peak**	**AUC** **(95% CI)**	0.620 (0.403–0.838)	0.476 (0.241–0.711)	0.513 (0.266–0.760)	0.636 (0.401–0.871)	0.636 (0.418–0.854)	0.636 (0.401–0.871)
	**Sensitivity**	0.412	0.294	0.824	0.882	0.529	0.882
	**Specificity**	0.818	0.545	0.455	0.545	0.837	0.545
**AUC**	**AUC** **(95% CI)**	0.572 (0.353–0.792)	0.471 (0.237–0.704)	0.497 (0.239–0.756)	0.701 (0.484–0.917)	0.658 (0.438–0.877)	0.701 (0.484–0.917)
	**Sensitivity**	0.471	0.353	0.941	0.941	0.765	0.941
	**Specificity**	0.818	0.364	0.636	0.545	0.636	0.545
**Wash in**	**AUC** **(95% CI)**	0.492 (0.247–0.737)	0.663 (0.431–0.895)	0.599 (0.354–0.844)	0.519 (0.269–0.769)	0.497 (0.262–0.733)	0.663 (0.431–0.895)
	**Sensitivity**	0.882	0.765	0.882	0.176	0.294	0.765
	**Specificity**	0.364	0.536	0.545	0.545	0.455	0.536
**Wash out**	**AUC** **(95% CI)**	0.567 (0.314–0.819)	0.642 (0.415–0.868)	0.535 (0.296–0.773)	0.540 (0.316–0.764)	0.428 (0.189–0.667)	0.642 (0.415–0.868)
	**Sensitivity**	0.824	0.824	0.765	0.529	0.235	0.824
	**Specificity**	0.545	0.636	0.545	0.636	0.455	0.636
**Combined ve and ADC**	**AUC** **(95% CI)**						0.840 * **(0.645–1.000)**
	**Sensitivity**						**0.941**
	**Specificity**						**0.818**

CI = confidence interval; ADC = apparent diffusion coefficient; MK = mean kurtosis; Ktrans = volume transfer constant; Kep = reflux exchange rate; vp = fractional volume of plasma; ve = fractional volume of extravascular extracellular space; CBV = cerebral blood volume; TTP = time to peak; AUC = area under the curve. ROI_1_ represents the tumor core and ROI_2-5_ represent concentric shells around ROI_1_, with increasing distance to ROI_1._ Significant AUC values are bold and marked with an asterisk (*).

## Data Availability

In order to safeguard the confidentiality of the participants, the data pertaining to this study are currently withheld from public access. The data can be shared upon request.
